# A Key Comprehensive System for Biobehavioral Surveillance of Populations Disproportionately Affected by HIV (National HIV Behavioral Surveillance): Cross-sectional Survey Study

**DOI:** 10.2196/39053

**Published:** 2022-11-15

**Authors:** Dafna Kanny, Dita Broz, Teresa Finlayson, Kathryn Lee, Catlainn Sionean, Cyprian Wejnert, NHBS Study Group

**Affiliations:** 1 Behavioral and Clinical Surveillance Branch Division of HIV Prevention Centers for Disease Control and Prevention Atlanta, GA United States; 2 see Acknowledgments

**Keywords:** HIV, biobehavioral surveillance, men who have sex with men, persons who inject drugs, heterosexually active persons at increased risk for HIV infection, HIV risk, public health, surveillance, HIV prevention, HIV epidemic

## Abstract

**Background:**

The National HIV Behavioral Surveillance (NHBS) is a comprehensive system for biobehavioral surveillance conducted since 2003 in 3 populations disproportionately affected by HIV: gay, bisexual, and other men who have sex with men (MSM); people who inject drugs; and heterosexually active persons at increased risk for HIV infection (HET). This ongoing and systematic collection and analysis of data is needed to identify baseline prevalence of behavioral risk factors and prevention service use, as well as to measure progress toward meeting HIV prevention goals among key populations disproportionately affected by HIV.

**Objective:**

This manuscript provides an overview of NHBS from 2003 to 2019.

**Methods:**

NHBS is conducted in rotating, annual cycles; these 3 annual cycles are considered a round. Venue-based, time-space sampling is used for the MSM population. Respondent-driven sampling is used for people who inject drugs and HET populations. A standardized, anonymous questionnaire collects information on HIV-related behavioral risk factors, HIV testing, and use of prevention services. In each cycle, approximately 500 eligible persons from each participating area are interviewed and offered anonymous HIV testing.

**Results:**

From 2003 to 2019, 168,600 persons were interviewed and 143,570 agreed to HIV testing across 17 to 25 cities in the United States. In the fifth round (2017 to 2019), over 10,000 (10,760-12,284) persons were interviewed each of the 3 population cycles in 23 cities. Of those, most (92%-99%) agreed to HIV testing. Several cities also conducted sexually transmitted infection or hepatitis C testing.

**Conclusions:**

NHBS is critical for monitoring the impact of the Ending the HIV Epidemic in the United States initiative. Data collected from NHBS are key to describe trends in key populations and tailor new prevention activities to ensure high prevention impact. NHBS data provide valuable information for monitoring and evaluating national HIV prevention goals and guiding national and local HIV prevention efforts. Furthermore, NHBS data can be used by public health officials and researchers to identify HIV prevention needs, allocate prevention resources, and develop and improve prevention programs directed to the populations of interest and their communities.

## Introduction

More than 40 years into the public health response to HIV, tremendous progress to prevent HIV transmission and save lives has been made globally and in the United States. Today, the tools to eliminate HIV exist, yet effective health interventions are not reaching populations that have been marginalized and are experiencing disproportionate impact of HIV [1,2]. Key members of the population and their partners, including gay, bisexual, and other men who have sex with men (MSM) and people who inject drugs, remain disproportionately affected by HIV [3]. Furthermore, social deprivation and poverty continue to be associated with high rates of HIV [4,5]. Biobehavioral surveillance of populations disproportionately affected by HIV has been critical to monitoring HIV prevention efforts and identifying areas of need, and it will continue to inform HIV prevention efforts, including those of Ending the HIV Epidemic in the United States by 2030 [6].

In 2003, the US Centers for Disease Control and Prevention (CDC), in collaboration with state and local partners and other surveillance and methodology experts, developed the National HIV Behavioral Surveillance (NHBS) as a comprehensive system for conducting biobehavioral surveillance among populations disproportionately affected by HIV [7]. To assure successful implementation, NHBS is focused on building relationships with community members, the intended populations, and prevention providers who work with these populations. NHBS has been funded through a series of cooperative agreements with collaborating state and local health departments. Health departments eligible to participate in NHBS are among those whose jurisdictions include a metropolitan statistical area (MSA) or a metropolitan division with high prevalence of HIV. Funded health departments conduct project activities within specified MSAs or metropolitan divisions. The key objectives of NHBS are to describe and monitor HIV behavioral risk factors, HIV testing, use of prevention services, and prevalence and trends in HIV infection in 3 populations disproportionately affected by HIV: MSM, people who inject drugs, and heterosexually active persons at increased risk for HIV infection (HET).

Male-to-male sexual contact is the most commonly reported route of HIV transmission in the United States, accounting for more than two-thirds of new diagnoses of HIV infection [3]. People who inject drugs are at high risk for HIV through sharing needles, syringes, or other drug injection equipment and through sexual contact. In the United States, about 1 in 10 HIV infections diagnosed are attributed to unsafe injection drug use or male-to-male sexual contact among people who inject drugs [8]. Among people who inject drugs, three-quarters of those who received a diagnosis of HIV infection live in urban areas [9]. About a quarter of new HIV diagnoses in the United States are associated with heterosexual sex [3]. Low-income HET in urban areas have highest HIV burden [10,11]. Stigma and discrimination related to male-male sex and drug use and overall health disparities linked with social and economic disadvantages make the populations surveyed in NHBS susceptible to multiple physical and health problems and can affect whether they seek HIV testing, treatment, and other health services [12-16]. Active community recruitment in NHBS ensures that impactful data are collected to inform prevention efforts for these populations and monitor progress. This manuscript provides an overview of NHBS from 2003-2019 focusing on the MSM, people who inject drugs, and HET populations.

## Methods

### Participants

HIV behavioral surveillance has been conducted in rotating, annual cycles since 2003 in populations disproportionately affected by HIV: MSM cycle [17], people who inject drugs cycle [18], and HET cycle [11]. For the HET cycle, NHBS considers poverty a qualifying risk factor for HIV infection. Specifically, participants are considered to have met the HET definition if they have income at or below 150% of the federal poverty level, adjusted for geographic cost of living differences. Participants in the HET cycle are asked about their combined monthly or yearly household income (in US $) from all sources for the calendar year before interview. Poverty is determined by using the US Department of Health and Human Services poverty guidelines. Because the poverty guidelines are not defined for the territory of Puerto Rico, the guidelines for the contiguous states and Washington, DC, are used for this jurisdiction. These 3 annual cycles are considered a round. In addition to the core cycles, a limited number of project areas had the option of conducting surveys in other key populations affected by HIV. In 2015, NHBS sampled young MSM aged 13 to 18 years in 3 project areas (NHBS-YMSM) [19]. In 2019-2020, NHBS received funding from the Secretary’s Minority AIDS Initiative Fund to conduct a pilot program to collect data among transgender women (NHBS-Trans) in 7 project areas [20]. All participants provide their informed consent to take part in the interview, HIV testing, specimen storage (eg, dry blood spots), and if applicable, other testing (eg, hepatitis, sexually transmitted infection [STI]). Participants must consent to the survey to be eligible for the other components; however, if participants do not consent to the survey but still wish to receive HIV testing or other testing, project staff in each NHBS project area will provide referrals and information for the person to access these resources.

### Ethics Approval

Activities for NHBS are approved by the CDC; NHBS is reviewed annually and determined to be a routine disease surveillance activity and thus exempt from ongoing CDC institutional review board (IRB) review (45 CFR § 46.102(l)(2)). Copy of this determination is provided in the NHBS protocol [21]. This project determination also covers secondary analyses of collected data and evaluation of NHBS, which is conducted on an ad hoc basis. These evaluations may include surveillance evaluations, program evaluations, and evaluation activities such as inclusion of different populations (eg, transgender persons, sex partners of MSM, people who inject drugs, or HET) or different cities (eg, Southern MSAs, which are not eligible for NHBS but have a high prevalence of HIV among heterosexuals). NHBS is also reviewed by applicable local IRBs in each participating project area.

NHBS is covered under the Assurance of Confidentiality for HIV data. NHBS data are anonymous. Participants are not required to provide their names or other personal identifiers as a condition for participation. To prevent inadvertent linkage, consent forms that must be signed (due to local IRB requirement) are not labeled with a survey ID number and are maintained separately from other documents. Blood specimens, lab slips, coupons, and questionnaires are linked by survey ID numbers only. As a component of CDC HIV surveillance, NHBS data are protected by the Assurance of Confidentiality (Section 308[d] of the Public Health Service Act, 42 US Code § 242 m[d]). This assurance prohibits the disclosure of any information by the CDC that could be used to identify individuals directly or indirectly. Data collection, management, and analysis for this project are conducted in compliance with the CDC’s Data Security and Confidentiality Guidelines for HIV, Viral Hepatitis, Sexually Transmitted Disease, and Tuberculosis Programs: Standards to Facilitate Sharing and Use of Surveillance Data for Public Health Action [22].

It is the responsibility of the CDC NHBS Publications Workgroup to facilitate the analysis and dissemination of NHBS data. NHBS data sets that contain aggregated data for all participating MSAs for a given cycle are maintained by the CDC. The NHBS Publications Workgroup has developed guidance to establish the methods for proposing and evaluating NHBS data analyses so that investigators can fairly participate in the process of publishing findings. All analyses of these multisite data sets must occur on the CDC premises in Atlanta, GA, or on the premises of a currently funded NHBS health department where they are housed.

### Study Design

NHBS cycles are repeated cross-sectional surveys of persons disproportionately affected by HIV. The survey methods used to recruit participants are venue-based sampling (VBS) and respondent-driven sampling (RDS). VBS and RDS have been found effective for recruiting populations that are hidden. Hidden populations are those for which no sampling frame exists or whose members engage in stigmatized or illegal activities, making them reticent to divulge information that may compromise their privacy. VBS recruits attendees of MSM-focused venues (eg, clubs, organizations, street locations) within the project area to obtain the desired sample and is used in the MSM cycles [23]. RDS is a chain recruitment method that begins with a set of seeds who recruit members of their social networks to participate in project activities, who in turn recruit other members of their social networks. RDS is used in the people who inject drugs, HET, and Trans cycles [24]. YMSM used 3 sampling methods: VBS, RDS, and Facebook sampling, which used targeted banner ads to identify and recruit YMSM [19].

### Procedures and Data Collection

NHBS activities are described in annual HIV surveillance reports and model protocols [21,25-27]. Trained interviewers use a standardized, anonymous questionnaire to collect information on HIV-related behavioral risk factors, HIV testing, and the use of HIV prevention services [28]. In each cycle, approximately 500 eligible persons from each participating project area are interviewed and offered optional, anonymous HIV testing. For each cycle, general NHBS eligibility criteria include age of 18 years or older, residence in participating MSA, no previous participation during the current survey cycle, ability to complete the survey in either English or Spanish, and ability to provide informed consent. In the past 5 rounds, for the MSM cycles, additional eligibility criteria included male sex at birth, male gender identity, and ever had oral or anal sex with a man. For the people who inject drugs cycles, additional eligibility criteria included injected drugs in the past 12 months and physical signs of recent injection or knowledge of injection. For the HET cycles, additional eligibility criteria included identify as male or female, had one or more opposite sex partner in the past 12 months, and aged 18 and 60 years.

There are 3 phases for NHBS implementation repeating annually. Every cycle starts with about 5 months (January to May) of formative assessment that includes interviews with people with lived experience and others closely knowledgeable about the populations [29,30]. Formative assessment helps project areas refine and develop their methods and operations for recruitment and data collection. Project areas often use formative assessments to answer key implementation questions, such as the appropriate incentive for participation, a safe, conveniently located field site location for data collection in RDS cycles, or identification of venues in the MSM cycle. The formative assessment also helps build community support for the survey. Formative assessment methods include a review of existing data, reports, and publications; qualitative interviews with key community partners, including service providers and community key informants; and ethnographic observations. From June to November, project areas collect biobehavioral data using different strategies to implement recruitment and data collection [31]. For MSM cycles, each project area conducts recruitment events at or near venues frequented by MSM. For people who inject drugs and HET cycles, project areas conduct recruitment and data collection at established field sites (eg, rented storefront, mobile van parked in an established location). In December, project areas begin closing out their projects.

### Questionnaire

The NHBS interview uses a standardized, anonymous questionnaire that takes 30 to 40 minutes to complete on average [28]. Eligible individuals who consent complete an interviewer-administered, standardized, in-person anonymous questionnaire using portable computers, such as laptops or tablets. NHBS uses a single instrument for each cycle in a round. With few exceptions (eg, cycle-specific eligibility criteria), the NHBS questionnaire uses the same standardized items for all 3 cycles to assess demographics and key indicators in the following domains: sexual behaviors, alcohol use, injection and noninjection drug use, HIV testing experiences, history of sexually transmitted diseases and hepatitis, social determinants or social conditions, and prevention activities, including pre-exposure prophylaxis. In accordance with the Paperwork Reduction Act, the Office of Management and Budget has approved the NHBS questionnaire [32]. For each round, the NHBS questionnaire is updated as needed based on feedback from interviewers, partners, and input from subject matter experts and experts in survey design. Project areas have an option to ask locally relevant questions for up to 10 additional minutes after the NHBS interview.

### HIV Testing

All participants are offered HIV testing regardless of their self-reported HIV status. Testing methods include conducting a rapid test to screen for infection. If this rapid test is positive, a follow up lab-based test or a different type of rapid test to confirm infection is used. Participants are given the option of receiving their rapid test result after completing the questionnaire. Appropriate risk-reduction counseling is provided to all participants who elect testing for HIV. Counselors tailor prevention messages to specific risks identified during the behavioral surveillance interview. Counselors provide referrals for treatment and other health and social services identified during the counseling session. All laboratory tests conducted in the United States used to diagnose infection are regulated by Clinical Laboratory Improvement Amendments (CLIA). Project areas select tests from a list of CLIA-waived HIV rapid tests, which are diagnostic tests approved for use in field settings by nonlaboratory staff.

### Additional Biological Testing

CDC’s Division of HIV Prevention has established collaborations with other divisions and agencies to fund additional biological testing as part of NHBS in select project areas [33]. These include collaboration with the CDC’s Division of STD Prevention on sexually transmitted infection (STI) testing [34] for (1) gonorrhea (Neisseria gonorrhoeae) and chlamydia (Chlamydia trachomatis) at the pharynx and rectum offered to MSM in 5 project areas in 2017 [35], (2) gonorrhea and chlamydia testing at the pharynx and vagina offered to young heterosexually active females aged 18 to 30 years in 5 project areas in 2019, and (3) pharyngeal, rectal, and urogenital gonorrhea and chlamydia testing offered to transgender women in 5 project areas in 2019-2020. All specimens were self-collected via swabs or urine in nonclinical settings. Additionally, in 2018, Division of HIV Prevention collaborated with the National Institutes of Health’s National Institute on Drug Abuse [36] and CDC’s Division of Viral Hepatitis [37] to provide hepatitis C virus (HCV) testing to people who inject drugs in 10 NHBS project areas [38]. Blood-based rapid HCV testing in the field and laboratory HCV RNA testing was offered to all people who inject drugs participants in the 10 project areas, results were provided to participants within 2 weeks of testing, and participant were referred to applicable care and treatment. In addition to the HIV testing offered as part of NHBS, project areas could conduct other testing with local funds if local regulations permit anonymous testing. Results of all biological testing conducted as part of NHBS are paired with the interview.

### Incentives

Participants are offered incentives in exchange for their participation, both for the interview and for HIV testing. If additional testing are offered, such as STI and HCV, participants are also offered incentives. Participants may receive incentive payments in person (eg, cash, gift card). Participant compensation for incomplete surveys may be offered in accordance with local policies. Incentives are given to those interviewed and tested for HIV (approximately $25 for each). In cycles using RDS, additional rewards (approximately $10) are paid to those who successfully recruit others. Additional incentives are generally provided for any additional testing (eg, HBV, HCV, STI). Local project areas determine the exact amount and type of incentives deemed appropriate for the local populations being interviewed and tested.

## Results

**Figure 1:**
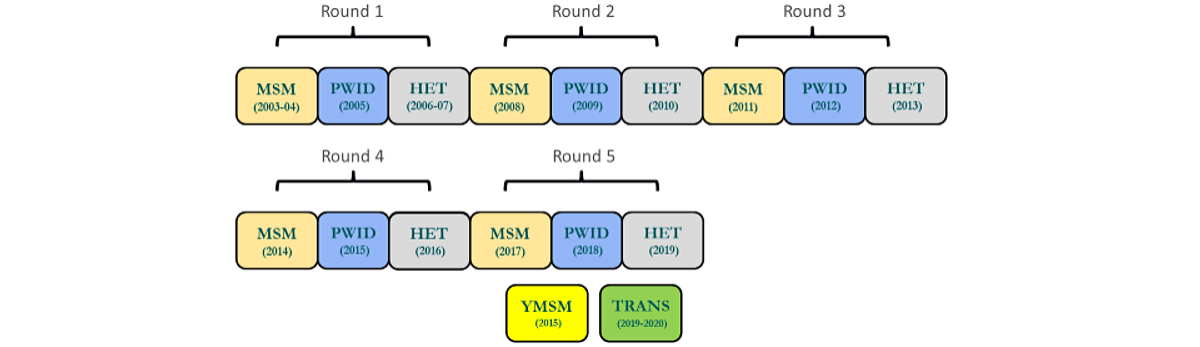
National HIV Behavioral Surveillance core and additional populations, 2003-2019. MSM: gay, bisexual, and other men who have sex with men; PWID: persons who inject drugs; HET: heterosexually active persons at increased risk for HIV infection; YMSM: young men who have sex with men; Trans: transgender women.

From 2003 to 2019, 5 rounds of NHBS were conducted (Figure 1). The number of completed interviews, HIV testing, STI testing, and HCV testing for each cycle between 2003 to 2019 are presented in Table 1. Overall, from 2003 to 2019, 168,600 persons were interviewed and 143,570 agreed to HIV testing. The fifth round was conducted from 2017 to 2019 in 23 MSAs (Table 2) [39], which represented 59% of all persons living with diagnosed HIV in urban areas with a population of at least 500,000 at the start of the funding cycle (year’s end 2016). In each cycle of the last round over 10,000 persons were interviewed (range 10,760-12,284), and of those interviewed, 33,433 HIV testing were completed (92%-99%). Several NHBS project areas conducted STI or HCV testing.

Additional rounds of NHBS are ongoing. The sixth round of NHBS was planned to start in 2020; however, due to the COVID-19 pandemic, NHBS data collection in 2020 was disrupted. Thus, the MSM cycle was repeated in 2021. The people who inject drugs cycle is conducted in 2022. Round 7 is scheduled to resume with routine cycle implementation in 2023. Since 2003, NHBS data have been used in over 400 peer-reviewed manuscripts authored by CDC, local project areas, and collaborators [40]. Local and aggregate level NHBS data have also been disseminated through surveillance reports and infographics [41] and scientific, community, and internal presentations.

**Table 1:** Project areas, records, HIV testing, sexually transmitted infection testing, and hepatitis C virus testing by population/cycle, National HIV Behavioral Surveillance, 2003-2019.

Year	Population/cycle	Number of project areas	Total number of records^a^	HIV testing^b^, n (%)	STI^c^ testing, n (%)	HCV^d^ testing, n (%)
2003-05	MSM^e^	15	10,030	2150 in 5 cities (36.7)	—^f^	—
2005	PWID^g^	22	11,613	—	—	—
2006-07	HET^h,i^	24	18,278	17,553 (96.0)	—	—
2008	MSM	21	9874	8654 (87.6)	—	—
2009	PWID	20	10,256	10,144 (98.9)	—	—
2010	HET	21	10,933	10,851 (99.2)	—	—
2011	MSM	20	9819	8922 (90.9)	—	—
2012	PWID	20	10,171	10,056 (98.9)		1461 in 4 cities (81.0)
2013	HET	20	10,535	10,479 (99.5)	—	—
2014	MSM	20	10,369	9384 (90.5)	—	—
2015	PWID	20	10,487	10,402 (99.2)	—	—
2015	YMSM^j^	3	569	508 (89.3)	—	—
2016	HET	17	9541	9445 (99.0)	—	—
2017	MSM	23	10,760	9888 (91.9)	2075^k^ in 5 cities (83.1)	—
2018	PWID	23	11,444	11,355 (99.2)	—	5190 in 10 cities (99.5)
2019	HET	23	12,284	12,190 (99.2)	456^k^ in 5 cities among women aged 18-30 (93.1)	—
2019-20	Trans^l^	7	1637	1589 (97.1)	824^k^ in 5 cities (90.4)	—
Total	—	—	168,600	143,570	3355	6651

^a^Total number of records in each cycle's analysis is harmonized across the years within a cycle and includes the number of records that were eligible, consented to the survey, completed the interview, and provided valid answers.

^b^Valid rapid or enzyme immunoassay test for HIV antibodies.

^c^STI: sexually transmitted infection.

^d^HCV: hepatitis C virus.

^e^MSM: gay, bisexual, and other men who have sex with men.

^f^Not collected.

^g^PWID: persons who inject drugs.

^h^HET: heterosexually active persons at increased risk for HIV infection.

^i^The first HET cycle was a pilot of the optimal operational definition of HET at increased risk for HIV as well as the optimal sampling strategy (venue-based sampling vs respondent driven sampling) to reach them. The first HET cycle was also the first population and cycle that HIV testing was offered in all project areas.

^j^YMSM: young men who have sex with men.

^k^At least one valid test for gonorrhea or chlamydia from pharyngeal swabs (all cycles), rectal swab (MSM and Trans cycles only), vaginal swab (HET cycle only), or urine specimen (Trans cycle only).

^l^Trans: transgender women.

**Table 2:** Participating project area (funded health department), by round and cycle, National HIV Behavioral Surveillance, 2003-2019.

	Round 1, 2003-2007			Round 2, 2008-2010			Round 3, 2011-2013			Round 4, 2014-2016			Round 5, 2017-2019		
	MSM1^a^	PWID1^b^	HET1^c^	MSM2	PWID2	HET2	MSM3	PWID3	HET3	MSM4	PWID4	HET4	MSM5	PWID5	HET5
Atlanta (Georgia Dept of Human Resources)	x^d^	x	x	x	x	x	x	x	x	x	x	x	x	x	x
Baltimore (Maryland Dept of Health and Mental Hygiene)	x	x	x	x	x	x	x	x	x	x	x		x	x	x
Boston (Massachusetts Dept of Public Health)	x	x	x	x	x	x	x	x	x	x	x	x	x	x	x
Chicago (Chicago Dept of Public Health)	x	x	x	x	x	x	x	x	x	x	x	x	x	x	x
Dallas (Texas Dept of Health)	x	x	x	x	x	x	x	x	x	x	x	x	x	x	x
Denver (Colorado Dept of Public Health)	x	x	x	x	x	x	x	x	x	x	x	x	x	x	x
Detroit (Michigan Dept of Community Health)		x	x	x	x	x	x	x	x	x	x	x	x	x	x
Fort Lauderdale (Florida Dept of Health)	x	x	x												
Houston (Houston Dept of Health and Human Services)	x	x	x	x	x	x	x	x	x	x	x	x	x	x	x
Indianapolis (Indiana State Dept of Health)															
Las Vegas (Nevada Dept of Health)		x	x												
Los Angeles (Los Angeles County Health Dept)	x	x	x	x	x	x	x	x	x	x	x	x	x	x	x
Memphis (Tennessee Dept of Health)												x	x	x	x
Miami (Florida Dept of Health)	x	x	x	x	x	x	x	x	x	x	x	x	x	x	x
Nassau (New York State Dept of Health)		x	x	x	x	x	x	x	x	x	x	x	x	x	x
New Haven (Connecticut Dept of Public Health)		x	x												
New Orleans (Louisiana Dept of Human Services)		x	x	x	x	x	x	x	x	x	x	x	x	x	x
New York City (NYC Dept of Health and Mental Hygiene)	x	x	x	x	x	x	x	x	x	x	x	x	x	x	x
Newark (New Jersey Dept of Health and Senior Services)	x	x	x	x	x	x	x	x	x	x	x	x	x	x	x
Norfolk (Virginia Dept of Health)		x	x									x	x	x	x
Philadelphia (Philadelphia Dept of Public Health)	x	x	x	x	x	x	x	x	x	x	x	x	x	x	x
Portland (Oregon Health Authority)												x	x	x	x
San Diego (California Dept of Health Services)	x	x	x	x	x	x	x	x	x	x	x	x	x	x	x
San Francisco (San Francisco Dept of Public Health)	x	x	x	x	x	x	x	x	x	x	x	x	x	x	x
San Juan (Puerto Rico Health Dept)	x	x	x	x	x	x	x	x	x	x	x	x	x	x	x
Seattle (Washington Dept of Health)		x	x	x	x	x	x	x	x	x	x	x	x	x	x
St Louis (Missouri Dept of Health and Senior Services)		x	x	x	x	x									
Washington DC (DC Dept of Health)	x		x	x	x	x	x	x	x	x	x	x	x	x	x
Total project areas	17	24	25	21	21	21	20	20	20	20	20	22	23	23	23

^a^MSM: gay, bisexual, and other men who have sex with men.

^b^PWID: people who inject drugs.

^c^HET: heterosexually active persons at increased risk for HIV infection.

^d^x: indicates the specific round and cycle in which the project areas participated.

## Discussion

### Principal Findings

NHBS data have been used to provide behavioral and community context for trends seen in HIV diagnoses reported to the CDC’s National HIV Surveillance System [42]. NHBS data have also described populations with high burden of HIV and thus have provided indications for intervention to prevent HIV transmission. Given the high levels of stigma, discrimination, and health inequity experienced by populations included in NHBS, this system provides data to address systemic and structural factors of HIV disparities. Through systematic, ongoing surveillance in groups disproportionately affected by HIV, NHBS has provided important information for planning and assessing efforts to prevent HIV in key populations.

NHBS populations often experience myriad comorbidities beyond HIV. To better serve these populations and assure successful implementation, NHBS seeks and maintains extensive collaborations. These collaborations include building relationships with community members, the intended populations, and prevention providers that work with these populations throughout the life cycle of the surveillance system. CDC and collaborators meet annually following data collection to debrief on methodological lessons learned in the preceding year and incorporate these into future iterations of NHBS. Collaborations to conduct additional biological testing that expands its public health mission beyond HIV have provided testing and referral to care for chlamydia, gonorrhea, and hepatitis C virus. These data have enhanced our knowledge of STIs and hepatitis C among NHBS populations, especially persons who may not access medical care [35]. Further, data gathered during these activities have addressed gaps in information about the prevalence of acute and chronic HCV infection among people who inject drugs in the United States [38].

Although HIV behavioral surveillance data cannot be used to evaluate the efficacy of specific interventions, these are important for monitoring whether HIV prevention efforts are reaching populations disproportionately affected by HIV within a community and whether these efforts meet local and national prevention goals. At the individual level, NHBS participants have benefited directly from HIV prevention counseling, knowledge of their HIV status, and referrals for additional HIV prevention information and linkage to care. Participants who have preliminary HIV-positive or confirmed HIV-positive test results were counseled and referred for treatment and case management services.

### Limitations

NHBS is not nationally representative and might not be generalizable to all US urban areas, nonurban areas, or all MSM, people who inject drugs, or HET populations. However, the hidden and hard-to-reach nature of these populations prevents collection of nationally representative samples. NHBS data represent the gold standard of national level data used to inform HIV prevention among these population in the United States. There are several sources of bias in RDS: (1) groups that are more insular (ie, more likely to recruit only within their own group) are more likely to be overrepresented (if recruitment chains become trapped inside the group) or underrepresented (if recruitment chains cannot access the group) in the sample than less insular groups; (2) groups with larger networks may be overrepresented in the sample because more recruitment paths lead to their members; and (3) some groups may be less willing or able to participate in the survey and would be underrepresented in the sample. There are several ways to assess this bias and compensate for it. Some of the potential sources of bias were controlled by NHBS project area staff; for instance, staff are encouraged to ensure that their initial peer recruits, or seeds, are diverse by race/ethnicity, gender, age, geographic location, and other important factors that would have the effect of increasing the insularity of recruitment and of homophily (ie, groups that recruit only within their own group). Project areas also implement lessons learned during formative assessment to mitigate potential participation bias. For example, information from formative assessment is used to optimize location and setup of field sites to ensure all population members have safe, convenient access to participants [43,44]. If necessary, multiple field sites are used.

Other sources of bias are considered during data analysis using information obtained during the survey. To calculate the population estimates and sample variances derived from RDS, participants’ network size and information on who recruited whom (made possible through the coupon tracking system) are factored in to arrive at population estimates that reflect the underlying population. If these sources of bias cannot be satisfactorily controlled and measured, or if there are unknown barriers to peer recruitment, some assumptions on which RDS is based may not be met and the resulting estimates may not reflect the true population parameters of the NHBS population. Formative assessment and monitoring the sample throughout data collection is critical to minimize the effect of these sources of bias.

Findings from venue-based sampling methods can only be generalized to venue-attending MSM [17,45]. Some persons who are otherwise eligible (eg, by age, sexual behavior, and residence) may not attend the venues eligible for NHBS operations during the data collection cycle or not attend venues at all. To minimize the effect of this bias, formative assessment is conducted throughout the data collection period to update venue and daytime periods. If new venues or daytime periods are identified or become accessible, they should be added to the sampling frames. Similarly, if a venue becomes inaccessible (eg, lost owner approval for NHBS operations) or ineligible (eg, venue closure), it should be removed from the venue frame. Despite these limitations, venue-based sampling has obtained large and diverse samples in other studies, including earlier cycles of NHBS.

Biases in enrollment and agreement to HIV testing may result in over- or underestimation of HIV prevalence or incidence. If those who agree to be tested differ from those who decline in terms of age, race/ethnicity, or sex, findings may be less generalizable.

### Conclusion

NHBS contributes to the nation’s program of HIV surveillance by being the only multisite system that provides estimates on key HIV prevention measures among populations disproportionately affected by HIV, including HIV-negative individuals. NHBS data provide valuable information for monitoring and evaluating national HIV prevention goals and for guiding national and local HIV prevention efforts. Furthermore, NHBS data can be used by public health officials and researchers to identify HIV prevention needs, allocate prevention resources, and develop and improve prevention programs directed to the populations of interest and their communities.

## Abbreviations

CDC: Centers for Disease Control and Prevention
CLIA: Clinical Laboratory Improvement Amendments
HCV: hepatitis C virus
HET: heterosexually active persons at increased risk for HIV infection
IRB: institutional review board
MSA: metropolitan statistical area
MSM: gay, bisexual, and other men who have sex with men
NHBS: National HIV Behavioral Surveillance
RDS: respondent-driven sampling
STI: sexually transmitted infection
Trans: transgender women
VBS: venue-based sampling
YMSM: young men who have sex with men
